# Health risk assessment of exposure to toxic elements resulting from consumption of dried wild-grown mushrooms available for sale

**DOI:** 10.1371/journal.pone.0252834

**Published:** 2021-06-23

**Authors:** Karolina Orywal, Katarzyna Socha, Patryk Nowakowski, Wojciech Zoń, Piotr Kaczyński, Barbara Mroczko, Bożena Łozowicka, Maciej Perkowski

**Affiliations:** 1 Department of Biochemical Diagnostics, Medical University of Bialystok, Faculty of Pharmacy with the Division of Laboratory Medicine, Bialystok, Poland; 2 Department of Bromatology, Medical University of Bialystok, Faculty of Pharmacy with the Division of Laboratory Medicine, Bialystok, Poland; 3 Department of Public International Law and European Law, University of Białystok, Faculty of Law, Białystok, Poland; 4 Institute of Plant Protection–National Research Institute, Bialystok, Poland; Universidade de Lisboa Instituto Superior Tecnico, PORTUGAL

## Abstract

Mushrooms exhibit a high ability to accumulate potentially toxic elements. The legal regulations in force in the European Union countries do not define the maximum content of elements in dried wild-grown mushrooms. This study presents the content of mercury (Hg), lead (Pb), cadmium (Cd) and arsenic (As) determined in dried wild-grown mushrooms (*Boletus edulis* and *Xerocomus badius*) available for sale. Moreover, the health risk associated with their consumption is assessed. The inductively coupled plasma mass spectrometry (Cd, Pb, As) and atomic absorption spectrometry (Hg) were used. The mean Hg, Cd, Pb and As concentration in *Boletus edulis* was 3.039±1.092, 1.983±1.145, 1.156±1.049 and 0.897±0.469 mg/kg and in *Xerocomus badius* 0.102±0.020, 1.154±0.596, 0.928±1.810 and 0.278±0.108 mg/kg, respectively. The maximum value of the hazard index (HI) showed that the consumption of a standard portion of dried *Boletus edulis* may have negative consequences for health and corresponded to 76.2%, 34.1%, 33% and 4.3% of the maximum daily doses of Hg, Cd, Pb and As, respectively. The results indicate that the content of toxic elements in dried wild-grown mushrooms should be monitored. The issue constitutes a legal niche where unfavourable EU regulations may pose a threat to food safety and consumer health.

## 1. Introduction

The impact of environmental pollution on human health is a common topic that has aroused interest for many decades, and the issue of food safety seems to be of particular importance. Food products should not only provide nutrients but should also be of appropriate health quality. Consumers should be confident that the products allowed for sale are completely safe and their consumption will not cause adverse health effects.

Poland is one of the largest exporters of mushrooms in Europe, growing about 300,000 tons of cultivated and forest mushrooms annually [1]. Picking forest mushrooms is a Polish autumnal tradition and the most popular method of their processing is drying. According to Statistics Poland, in the last decade, the volume of forest mushrooms sold has fluctuated from 2,599 tons (in 2015) to 7,302 tons (in 2017). In 2018, 159 tons of the king boletus and 864 tons of the chanterelle have been exported from Poland [2]. Mushrooms are offered for sale to retail customers in large-format chains, which makes them widely available. The sales are also boosted by mass media campaigns promoting a healthy diet based on products of natural origin.

The fruits of the forest floor have been appreciated for centuries as a source of food which has nutritional and health-promoting properties. Due to their specific aroma and taste, mushrooms are a valued product, used in cuisines throughout the world. They are regarded as a functional food as they contain highly nutritious proteins, a composition of vitamins and minerals, and because they are low in fat [3]. Moreover, they provide relatively little energy and are considered to be a source of dietary fiber, mostly in insoluble form, and polyunsaturated fatty acids [4]. Wild-grown mushrooms contain minerals essential for humans, such as potassium, phosphorus, magnesium, calcium, sodium, zinc, copper, manganese, nickel, selenium, and cobalt, in varying concentrations, depending on the species [5]. The content of biologically active compounds in mushrooms makes them beneficial to humans. They might have the potential to prevent malignant diseases, hypercholesterolemia, diabetes, hypertension, inflammation, liver dysfunction, viral and bacterial infections or cardiovascular diseases [6–9].

The development of industry and transport, as well as improper waste management contribute to significant amounts of pollution, among which toxic trace elements are of crucial importance. Mushrooms have a strong capacity to absorb potentially toxic trace elements from soils, including mercury (Hg), lead (Pb), cadmium (Cd), arsenic (As), accumulate them in their bodies and their concentrations in mushrooms can exceed the levels found in crops, fruit and vegetables [10–12].

The most important sources of soil pollution with toxic metals are industrial and transport emissions, agriculture, surface runoff from roads and waste storage. These trace elements have a harmful effect on human health, the strength of which depends on the age at which exposure occurred and its duration. Toxic elements can accumulate in organs and tissues, which is conditioned by their rapid absorption and slow excretion from the human body. Therefore, the health effects of regular consumption of products containing heavy metals may become manifest after many years [13]. Pb has a highly toxic influence, stemming from its action on the immune, nervous, urinary and cardiovascular systems. The main pathomechanism is associated with the induction of oxidative stress, leading to generation of reactive oxygen species. Pb exposure causes a number of chromosomal mutations, disorders in DNA repair and, in consequence, initiation of carcinogenesis [14]. Another toxic element, Cd, according to IARC (International Agency for Research on Cancer), belongs to group 1 of human carcinogens and the relationship between Cd exposure and tumour development has been confirmed in cancers of the kidney, liver, stomach, urinary bladder, prostate, pancreas and breast [15]. Similarly to Pb, Cd induces oxidative stress and irreversible damage to DNA because Cd exerts an inhibitory effect in the process of attaching repair proteins to damaged nucleotides. Moreover, exposure to Cd causes pathological changes to the bones, as well as the cardiovascular, urinary and endocrine systems [16]. As is also classified as a human carcinogen and exposure to its compounds is associated with malignant diseases of the skin, lungs and urinary bladder [15]. Moreover, chronic exposure to As causes chromosomal aberrations, oxidative stress, impairment of DNA repair and disruption of growth factor synthesis. The above-mentioned processes lead to the development of lung and liver diseases, neurological disorders, hypertension, anaemia, diabetes and ischemic heart disease [17, 18]. Hg is one of the most toxic elements on the periodic table, and methylmercury, due to its ability to accumulate in the human body, has a negative effect on the liver and the nervous, reproduction and cardiovascular systems [19]. This element easily crosses the blood-brain barrier, causing neurological disturbances. Exposure to Hg is associated with the development of amyotrophic lateral sclerosis, multiple sclerosis, Parkinson’s and Alzheimer’s diseases [20, 21]. Furthermore, Hg exposure may cause dysfunction of the cardiovascular, endocrine, reproductive and urinary systems [22, 23].

Most of the existing studies have been conducted on fresh mushrooms, while the popularity of dried products, which are available in all seasons, is steadily increasing. Moreover, the drying process does not cause loss of mushrooms’ nutritional value, but this also applies to the potentially toxic element contamination [24]. The regulations which are currently in force in the European Union countries define the maximum permissible content of Cd and Hg only in fresh mushrooms, in the case of Pb only in fresh cultivated mushrooms, while in the case of As, the maximum permissible content in mushrooms is not specified [25–30]. No legal regulations exist that define the maximum content of toxic elements in dried wild-grown mushrooms. The highest permissible levels of Pb for *Agaricus bisporus* (common mushroom), *Pleurotus ostreatus* (oyster mushroom), *Lentinula edodes* (shiitake mushroom) are established at 0.30 mg / kg of fresh matter. For the same species of fungi, the maximum level of Cd is also set: 0.20 mg / kg of fresh matter. For other mushroom species, the maximum level of Cd is 1.0 mg / kg. All the indicated maximum levels apply after washing of the mushrooms and separating the edible part. If certain maximum levels are applied to dried foodstuffs, changes in the concentration of contamination due to drying processes should be considered [25]. In the European Union, the legal maximum residue levels (MRLs) for Hg compounds (sum of Hg compounds expressed as Hg) is 0.05 mg / kg for cultivated fungi and 0.5 mg / kg for wild fungi, except for the boletus (*Boletus edulis*) with the MRL of 0.9 mg / kg. These levels apply to the entire product after removal of soil or substrate [30]. Therefore, it is worth asking the question about the safety of the consumption of natural products in the form of dried wild mushrooms.

This study aimed to update information on the content of toxic trace elements (Hg, Pb, Cd and As) accumulated in dried wild-grown mushrooms available for sale, indicating the safety or risk associated with their consumption.

## 2. Materials and methods

### 2.1. Materials

The material for research consisted of 80 samples of dried edible mushrooms, belonging to the most often consumed species in Poland: *Boletus edulis* (40 samples) and *Xerocomus badius* (40 samples). The mushrooms were purchased in 5 European chains of supermarkets, with 4–10 samples from separate batches for each species. According to the producers, they had been harvested in 2018–2019. All purchased mushrooms had been produced by Polish companies, and in most cases Poland was indicated as the country of origin of the mushrooms. The characteristics of studied samples of mushrooms is presented in Table 1.

**Table 1:** Characteristics of studied samples of mushrooms.

Species	Supermarket/City	Company	Number of batches
*Xerocomus badius*	Auchan/Białystok	PolGrzyb	5
*Xerocomus badius*	Auchan/Białystok	Tagros Polska	5
*Xerocomus badius*	Kaufland/Białystok	JamPol	10
*Xerocomus badius*	Lidl/Białystok	Nasza Chata	10
*Xerocomus badius*	Carrefour/Białystok	RunoPol	5
*Xerocomus badius*	Tesco/Białystok	RunoPol	5
*Boletus edulis*	Auchan/Białystok	PolGrzyb	4
*Boletus edulis*	Auchan/Białystok	Tagros Polska	6
*Boletus edulis*	Kaufland/Białystok	JamPol	10
*Boletus edulis*	Lidl/Białystok	Nasza Chata	10
*Boletus edulis*	Carrefour/Białystok	RunoPol	5
*Boletus edulis*	Tesco/Białystok	RunoPol	5

### 2.2. Sample preparation procedure

The mushroom samples were powdered using mechanical homogenizer (IKA Ultra-Turrax T18 digital, Staufen, Germany) and stored in polypropylene containers at -20°C until analysis. The determination of mercury concentration was performed directly in homogenized dried samples (0.05–0.1 g). To determine the concentration of Pb, Cd and As, powdered mushrooms (0.2–0.3 g) were mineralized with concentrated (69%) nitric acid (Tracepur, Merck, Darmstadt, Germany), using a closed-loop microwave system (Speedwave, Berghof, Königssee Germany) according to procedure described previously [31]. The mineralisation process included four steps with different temperatures (1^st^– 170°C, 2^nd^– 190°C, 3^th^– 210°C, 4^th^– 50°C) and pressure (1^st^– 20 atm, 2^nd^– 30 atm, 3^th^– 40 atm, 4^th^– 40 atm) for 10, 10, 10 and 18 min, respectively.

### 2.3. Determination of mercury concentration

Hg was determined using a Single-Purpose Atomic Absorption Spectrometer AMA-254 (AMA 254, Leco, Czech Republic). The procedure was performed according to manufacturer’s recommendations. The mushroom samples were placed in a cuvette in the analyzer chamber, dried and then burned at 600°C in an oxygen atmosphere, the Hg vapors being trapped by a gold amalgamator. The released Hg was measured in the following conditions: drying time– 60 s, decomposition time– 150 s, waiting time– 65 s. The limit of detection for Hg was 0.003 ng/sample. The concentrations of Hg in the samples were presented as μg /kg of dried mushrooms.

### 2.4. Determination of lead, cadmium and arsenic concentrations

Pb, Cd and As contents were determined by inductively coupled plasma mass spectrometry (ICP-MS) (NexION 300 D ICP-MS, PerkinElmer, USA) with kinetic energy discrimination chamber (KED) (in the case of As determination, this mode uses collisions and kinetic energy discrimination to correct polyatomic interferences), or using the standard method (in the case of Pb and Cd). The instrumental conditions are presented in Table 2. The limit of detection (3.3 times the standard deviation of the regression line divided by the slope of the calibration curve) was 0.16 μg/kg for Pb, 0.017 μg/kg for Cd and 0.019 μg/kg for As. The concentration of Pb, Cd and As in the samples were presented as μg/kg of dried mushrooms.

**Table 2:** ICP-MS conditions for As, Pb, Cd determination.

Parameter	Analytical conditions		
	As	Pb	Cd
Mode	KED	Standard	Standard
Mass (amu)	74.9216	205.975	109.903
		206.976	110.904
		207.977	112.905
			113.904
Dwell Time per amu (ms)	50		
Integration Time (ms)	1000		
Detector Calibration Mode	Dual		
Replicates	5		
Internal Standard	50 μg/L of indium in 2% HNO_3_		

KED–kinetic energy discrimination.

### 2.5. Accuracy check of the methods

Quality control was performed by analyzing certified reference material CS-M-3 Dried Mushroom Powder (Institute of Nuclear Chemistry and Technology, Poland). The certified material was subjected to the same pretreatment and analysis procedure as the studied samples. The reference material was analyzed every tenth studied sample. The certified content was 2.849±104 mg/kg of dried mass for Hg, 1.863±0.108 mg/kg of dried mass for Pb, 1.229±0.11 mg/kg of dried mass for Cd and 0.651±0.026 mg/kg of dried mass for As. All the results of quality control analysis were in the reference range provided by the manufacturer of the certified reference material. The recovery figures for the analytical methods used in estimation of Hg, Pb, Cd and As were 104%, 107%, 97.5% and 105%, respectively. The precision values of the methods for determination of Hg, Pb, Cd and As were 3.2%, 2.6%, 3.5% and 3.7%, respectively.

### 2.6. Human health risk assessment

To assess the carcinogenic and non-carcinogenic human health risk associated with the consumption of the studied mushrooms, the following indicators were calculated: EDI (Estimated Daily Intake), THQ (Target Hazard Quotient), CR (Carcinogenic Risk), HI (Hazard Index). Moreover, PTWI (Provisional Tolerable Weekly Intake) for Hg, PTMI (Provisional Tolerable Monthly Intake) for Cd and BMDL (Benchmark Dose Lower Confidence Limit) for As and Pb were used. The standard portion of fresh mushrooms consumed per person in Poland is 100 g and dry mass in mushrooms constitutes about 10% [32, 33]. In calculations of the above-mentioned indicators, we assumed that the average weight of a person was 70 kg and the standard portion of dried mushrooms consumed was 10g.

In 2011–2013, the Joint FAO/WHO Expert Committee on Food Additives (JECFA) established that PTWI for Hg is 4 μg/kg/week, PTMI for Cd is 25 μg/kg/month, BMDL for As is 3 μg/kg/day (lung cancer), 5.2 μg/kg/day (bladder cancer), 5.4 μg/kg/day (skin cancer) and BMDL for Pb is 0.5 μg/kg/day (neurotoxicity effect), 1.5 μg/kg/day (cardiovascular risk), 0.63 μg/kg/day (nephrotoxicity effect) [34–36]. On this basis, the tolerable daily intake of Hg, Pb, Cd and As was set as 0.57 μg/kg/day, 0.5 μg/kg/day, 0.83 μg/kg/day and 3 μg/kg/day, respectively.

The EDI is an index showing the transfer of elements to the human body through food consumption. It depends on the concentration of a given element and the amount of food consumed daily. EDI was determined according to the Eq (1):

EDI=FIRxC, (1)

where FIR is an average daily consumption of mushrooms by a person with an average body weight of 70 kg. According to published data, FIR is estimated as 27g/day for fresh mushrooms, which means 2.7g/day for dried mushrooms [37]. C- is a mean concentration of a given element in the studied sample.

The THQ is an index determining the potential non-carcinogenic health risk resulting from the consumption of contaminated food. It depends on the frequency and duration of exposure to toxic elements, daily food consumption, oral reference doses of the elements, body weight, the value of average exposure and the elements’ concentrations. If THQ>1, food consumption is perceived as hazardous to health, but if THQ≤1, a health risk is unlikely to occur [38]. THQ was determined according to the Eq (2):

$$THQ=10^{‐3}\;x\;(Efr\;x\;EDtot\;x\;FIR\;x\;C)/(RfDo\;x\;BWa\;x\;ATn),$$ (2)

where Efr is exposure frequency (365 days per year), EDtot is exposure duration (70 years–as estimated average life expectancy [39]), FIR is the calculated average daily consumption of dried mushrooms (2.7 g/day) [37], C is the average concentration of the toxic elements in the sample (mg/kg), RfDo is the oral reference dose specific for every element (Hg– 0.0003 mg/kg/day, Pb– 0.0035 mg/kg/day, Cd– 0.001 mg/kg/day and As– 0.0003 mg/kg/day [40], BWa is the average body weight (70 kg) and ATn is the average exposure in a year (365 days/year x 70 years).

The CR is an index determining the potential carcinogenic health risk resulting from the intake of carcinogens. The tolerable risk is when the value of CR is below 10^−4^ [41]. The CR was calculated according to the following Eq (3):

$$CR=10^{‐3}\;x\;(Efr\;x\;EDtot\;x\;EDI\;x\;CSf)/ATn,$$ (3)

where CSf is the oral slope factor for carcinogens, amounting to: 1.5 for As, 0.0085 for Pb and 6.3 mg/kg-day for Cd [42].

The HI index is the sum of hazard quotients for Pb, Cd and As and was calculated according to the Eq (4):

$$HI=\sum_{i=k}^{n}THQs,$$ (4)

where, THQs is the target hazard quotient estimated for individual elements. The HI value above 1 is considered unsafe for human health [43].

### 2.7. Statistical analysis

Statistical analysis was performed using Statistica software 13.0 (Statsoft, Krakow, Poland). The normality of the data was verified by means of the Shapiro-Wilk test and the Kolgomorov-Smirnov test. No criterion of normality was observed, therefore to calculate significant differences, the Kruskal-Wallis and Mann-Whitney U tests were performed. Differences were considered significant when p<0.05.

## 3. Results and discussion

### 3.1. The concentrations of Cd, Pb, As and Hg in dried mushrooms

The concentrations of Cd, Pb, As and Hg determined in dried mushrooms are presented in Table 3.

**Table 3:** Concentrations of Cd, Pb, As and Hg in dried mushrooms (mg/kg).

Mushroom species	*Boletus edulis* n = 40	*Xerocomus badius* n = 40	*Boletus edulis vs Xerocomus badius*
Cd content			
mean	1.983	1.154	p<0.001
median	1.638	0.975	
min.	0.551	0.486	
max.	5.212	2.750	
SD	1.145	0.596	
Q_1_	1.241	0.699	
Q_3_	2.602	1.378	
Pb content			
mean	1.156	0.928	p<0.05
median	0.794	0.486	
min.	0.327	0.228	
max.	4.700	1.167	
SD	1.049	1.810	
Q_1_	0.591	0.339	
Q_3_	1.167	0.857	
As content			
mean	0.897	0.278	p<0.001
median	0.745	0.243	
min.	0.433	0.096	
max.	2.449	0.578	
SD	0.469	0.108	
Q_1_	0.623	0.204	
Q_3_	0.958	0.350	
Hg content			
mean	3.039	0.102	p<0.001
median	2.944	0.100	
min.	1.201	0.058	
max.	5.394	0.163	
SD	1.092	0.020	
Q_1_	2.159	0.089	
Q_3_	3.773	0.109	

n- number of samples; SD-standard deviation; Cd-cadmium; Pb-lead; As- arsenic; Hg-mercury; Q_1_-lower quartile; Q_3_-upper quartile, p<0.05- statistically significant value.

Comparing the results of this research with the literature reports, it can be suggests that the content of toxic elements varies across mushroom species. Statistical analysis showed a significantly higher concentration of all the tested toxic elements in *Boletus edulis* compared to the values obtained in the samples of *Xerocomus badius*. In the case of *Xerocomus badius*, the concentration values of the analysed elements, arranged from the lowest to the highest, proved to be: Hg <As <Pb <Cd. The contents of heavy metals in *Boletus edulis* were different: As <Pb <Cd <Hg.

The analysis of Cd content in the tested dried mushrooms showed that the highest concentrations were found in *Boletus edulis*. In *Xerocomus badius*, the median Cd concentration was 0.975 mg/kg. On the other hand, the concentration of Cd in *Boletus edulis* was 1.638 mg/kg, which is a value higher by almost 170% compared to the amount found in *Xerocomus badius*. This difference is statistically significant and the obtained valuesindicate an increased accumulation of Cd by the species *Boletus edulis*. The obtained results are consistent with data presented by other researchers. Chiocchetti et al. found that Cd concentration in samples of dried mushrooms was 1.310 ± 0.052 mg/kg and that in cooked mushrooms it decreased to 0.756 ± 0.029 mg/kg [44]. In forest mushrooms collected in the area of Wysoczyzna Siedlecka, the concentration of Cd was found to be 0.746 mg/kg in the dry matter of *Xerocomus badius* and an almost 2.5 times higher concentration of this element was detected in *Boletus edulis* (1.840 mg/kg) [45]. On the other hand, Giannaccini et al. found a concentration of Cd in *Boletus edulis* reaching even 3.400 mg/kg dry weight, which is more than twice the value obtained in the studies carried out in this research [11]. The highest Cd concentration in *Xerocomus badius* described in the literature, reaching the value of 333 mg/kg dry weight, was found in mushrooms collected from a polluted area near a smelter in Lhota (Czech Republic) [46].

In *Xerocomus badius*, the median Pb concentration was 0.486 mg/kg. On the other hand, the concentration of Pb in *Boletus edulis* was 1.6 times higher (0.794 mg/kg). The demonstrated increase in the concentration of Pb in *Boletus edulis* is statistically significant in comparison with the value obtained in *Xerocomus badius*, which proves increased accumulation of Pb by the former species. In their studies, Adamiak et al. obtained an over 2 times lower Pb content in *Xerocoums badius* (0.234 mg/kg) and 1.5 times lower in *Boletus edulis* (0.513 mg/kg) [45]. Research carried out in Spain on samples of dried *Boletus edulis* showed even lower values of Pb level (0.094 mg/kg) [44]. On the other hand, Giannaccini et al. found that the median concentration of Pb in the dry matter of *Boletus edulis* was very high, reaching even 2.800 mg/kg [11].

The median As concentration in *Xerocomus badius* was 0.243 mg/kg–more than 3 times as low as that found in *Boletus edulis* (0.745 mg/kg). The difference in As content between these two species of mushrooms was statistically significant. Also, Adamiak et al. did not find any tendency of the fruiting bodies of *Boletus edulis* to excessively accumulate As compounds in their structure. However, consistent with the presented results, As content in *Xerocomus badius* was lower (0.182 mg/kg) than in *Boletus edulis* (0.282 mg/kg). However, a high content of this element, reaching the value of 0.791 mg/kg, was recorded in dried red-cappet bolete [45]. On the other hand, Chiocchetti et al. reported a very low As concentration in *Boletus edulis*, amounting to only 0.094 mg/kg [44]. The range of average As concentrations found in dried fruiting bodies of *Boletus edulis* from Europe was 0.1–1.1 mg/kg, while in mushrooms harvested in Mexico, As value was 7 times higher [47, 48].

The content of Hg in *Xerocomus badius* was the lowest of all the heavy metals tested and its median was 0.100 mg/kg. On the other hand, the highest Hg concentration was found in *Boletus edulis*, reaching 2.944 mg/kg. The amount of Hg in *Boletus edulis* is therefore almost 30 times higher than in *Xerocomus badius*, and the difference is statistically significant. Hg is actively accumulated by many species of mushrooms and its content in these products is a subject of research in many countries. Reported Hg concentrations in *Boletus edulis* from Poland, Italy and Spain are high and amount to 1.2 ± 1.4 mg/kg to 7.6 ± 3.1 mg/kg (range 0.02–14.0 mg/kg dry weight) in caps and 1.2 ± 0.7 mg/kg to 8.4 ± 7.4 mg/kg (range 0.05–22 mg/kg dry weight) in whole fruiting bodies [11, 49, 50]. The maximum Hg content in *Boletus edulis* from the Świętokrzyskie Mountains area was as high as 14 mg/kg of dry mass [47]. Such a high content of Hg in *Boletus edulis* was not recorded by Adamiak et al. The level of Hg found in this species of mushrooms was only 3 times higher (0.416 mg/kg) than that found in *Xerocomus badius* (0.133 mg/kg) [45]. On the other hand, in dried *Boletus edulis*, Chioccheti et al. found twice as high Hg concentrations, reaching 0.953 mg/kg [44]. It has been reported that concentration of Hg in mushrooms varied significantly depending on the species and fruiting bodies of *Boletus edulis* is characterized by a relatively high bioconcentration factors (BCF) value [51]. Extremely high Hg values in *Boletus edulis* (32 ± 19 mg/kg of dry mass) were detected in mushrooms from areas located in the vicinity of a mercury smelter and a copper smelter in Slovakia [52].

### 3.2. Health risk assessment

The presence of Cd, Pb, As and Hg in food is not desirable because prolonged exposure may have a toxic effect, causing the development of many disorders, including malignant diseases. The potential health risk resulting from the consumption of a standard portion of dried mushrooms was estimated based on EDI, THQ, CR and HI values, presented in Table 4.

**Table 4:** Estimated Daily Intake (EDI), Target Hazard Quotient (THQ), Carcinogenic Risk (CR) and Hazard Index for Cd, Pb, As and Hg, resulting from the consumption of dried mushrooms by a person with an average body weight of 70 kg.

	*Boletus edulis*			*Xerocomus badius*		
	Cd					
	EDI (mg/day)	THQ	CR	EDI (mg/day)	THQ	CR
mean concentration	5.35E-03	7.65E-02	3.37E-05	3.12E-03	4.45E-02	1.96E-05
min	1.49E-03	2.13E-02	9.38E-06	1.31E-03	1.87E-02	8.27E-06
max	1.41E-02	2.01E-01	8.87E-05	7.43E-03	1.06E-01	4.68E-05
	Pb					
	EDI (mg/day)	THQ	CR	EDI (mg/day)	THQ	CR
mean concentration	3.12E-03	1.27E-02	2.65E-08	2.51E-03	1.03E-02	2.13E-08
min	8.84E-04	3.61E-03	7.51E-09	6.17E-04	2.52E-03	5.24E-09
max	1.27E-02	5.18E-02	1.08E-07	3.15E-02	1.29E-01	2.68E-07
	As					
	EDI (mg/day)	THQ	CR	EDI (mg/day)	THQ	CR
mean concentration	2.42E-03	1.15E-01	3.64E-06	7.53E-04	3.59E-02	1.13E-06
min	1.17E-03	5.57E-02	1.76E-06	2.60E-04	1.24E-02	3.89E-07
max	6.61E-03	3.15E-01	9.92E-06	1.56E-03	7.43E-02	2.34E-06
	Hg					
	EDI (mg/day)	THQ	CR	EDI (mg/day)	THQ	CR
mean concentration	8.21E-03	3.91E-01	-	2.76E-04	1.31E-02	-
min	3.25E-03	1.55E-01	-	1.59E-04	7.57E-03	-
max	1.46E-02	6.94E-01	-	4.42E-04	2.11E-02	-
Hazard Index (Cd, Pb, AS)						
mean concentration	5.95E-01			1.04E-01		
min	3.02E-01			5.33E-02		
max	1.10E+00			2.16E-01		

Tolerable daily intake: Hg = 0.57 μg/kg/day, Pb = 0.5 μg/kg/day; Cd = 0.83μg/kg/day, As = 3μg/kg/day.

Reference values: THQ≤1; CR<10^−4^; HI<1.

The EDI of Cd for *Boletus edulis* and *Xerocomus badius* was 5.35E-03 mg/day and 3.12E-03 mg/day respectively, while the values for Pb were similar for both mushroom species (3.12E-03 mg/day for *Boletus edulis* and 2.51E-03 mg/day for *Xerocomus badius*). The values of the EDI of As in *Boletus edulis* (2.42E-03 mg/day) was higher than in *Xerocomus badius* (7.53E-04 mg/day). Also EDI of Hg was much higher for *Boletus edulis* (8.21E-03 mg/day) than for the other mushroom species (2.76E-04 mg/day).

The THQ index for the consumption of 2.7 g of dried mushrooms was calculated for a person with an average body weight of 70 kg. The value of THQ in ascending order in *Boletus edulis* was as follows: Cd (7.65E-02) < Pb (1.27E-02) < Hg (3.91E-01) < As (1.15E-01). The THQ indexes for *Xerocomus badius* were lower than for *Boletus edulis*: 3.59E-02 for As, 4.45E-02 for Cd, 1.03E-02 for Pb and 1.31E-02 for Hg. The THQ values for both mushroom species and for all the studied elements were below 1, which means there is no potential non-carcinogenic health risk associated with the consumption of these mushrooms.

The CR index of Cd ranged from 3.37E-05 for *Boletus edulis* to 1.96E-05 for *Xerocomus badius*, while the cancer risk of Pb was much lower (2.65E-08 for *Boletus edulis* and 2.13E-08 for *Xerocomus badius*). The cancer risk resulting from the ingestion of As was also similar in both mushroom species (3.64E-06 for *Boletus edulis* and 1.13E-06 for *Xerocomus badius*). The carcinogenic risk resulting from Hg consumption was not determined because Hg is not considered a direct carcinogen. The USEPA claims that CR<10^−6^ is not significant for human health. CR between 10^−6^ to 10^−4^ is tolerable for consumers and CR>10^−4^ is not acceptable and is associated with increased risk of cancer [53]. None of the studied mushrooms exceeded the CR index by more than 10^−4^ so there is no increased risk of malignant diseases resulting from the consumption of the studied dried mushrooms.

The HI reflects the risk for human health resulting from the accumulation of different toxic elements. The HI index had the following values: 5.95E-01 for *Boletus edulis* and 1.04E-01 for *Xerocomus badius*. When the HI is above 1, it indicates a significant health risk. It is noticeable that the maximum value of HI in *Boletus edulis* reached 1.10E+00, which means that the consumption of these mushrooms could have negative consequences for human health.

The toxicological risk of consuming a standard portion of dried mushrooms expressed as a % of the daily reference dose of each element, calculated for a person weighing 70 kg is presented in Table 5.

**Table 5:** Estimated contribution (%) in a daily toxic dose resulting from the consumption of a standard portion (10 g) of dried mushrooms by a person with an average body weight of 70 kg.

	*Boletus edulis*			*Xerocomus badius*		
	As					
	lung cancer	bladder cancer	skin cancer	lung cancer	bladder cancer	skin cancer
mean concen-tration	4.274	4.274	2.466	1.328	1.328	0.766
min	2.064	2.064	1.191	0.458	0.458	0.264
max	11.665	11.665	6.730	2.753	2.753	1.588
	Pb					
	neurotoxicity	cardiovascular diseases	nephrotoxicity	neurotoxicity	cardiovascular diseases	nephrotoxicity
mean concentration	33.049	33.049	26.229	26.539	26.539	21.063
min	9.350	9.350	7.420	6.525	6.525	5.179
max	134.292	134.292	106.581	333.570	333.570	264.738
	Cd					
	% TI					
mean concentration	34.135			19.875		
min	9.495			8.367		
max	89.709			47.339		
	Hg					
	% TI	nephrotoxicity		% TI	nephrotoxicity	
mean concentration	76.170	72.361		2.559	2.431	
min	30.123	28.617		1.476	1.402	
max	135.188	128.429		4.104	3.899	

TI–tolerable intake.

The consumption of 10 g of dried *Boletus edulis* provided 76% of the daily dose of Hg, reaching the maximum value of more than 135%, whereas the range for *Xerocomus badius* was much lower (1.4–4.1%). The percent of the daily dose of Cd was 1.5 times higher for *Boletus edulis* (range 9.5%-89.7%) than for *Xerocomus badius* (8.3–47.3%). The mean values of the daily dose of Pb in the case of *Boletus edulis* were equal to 33% of the doses causing neurotoxicity and increased risk of cardiovascular disease and 26% of the dose associated with nephrotoxicity. It is noticeable that for some samples of *Boletus edulis*, the daily doses of Pb were 134% for neurotoxicity and risk of cardiovascular disease and 106% for nephrotoxicity. The mean values of the daily dose of *Xerocomus badius* were lower (26% for neurotoxicity and risk of cardiovascular disease, 21% for nephrotoxicity) but the maximum values reached even 333% of the daily dose. The lowest values for both mushroom species concerned the daily doses of As (2.4–4.2% for *Boletus edulis* and 0.76–1.33% for *Xerocomus badius*).

### 3.3. The need for monitoring and clarifying legal regulations

Due to the toxicity of the tested substances, their presence in food products such as fungi is obviously undesirable. At the same time, an inconsistent approach to the presence of certain metals in mushrooms can be observed. As already mentioned, no permissible limits of As in mushrooms have been defined. In the case of lead, the European Union’s approach is quite conservative (standards have been indicated for only 3 species: *Agaricus bisporus*, *Pleurotus ostreatus* and *Lentinula edodes*). The level of Cd is specified for all fungi, although it is diversified (for the three above-mentioned species, the standards are much stricter than for other fungi). The concentration levels are also defined for Hg compounds, but differentiation has been made for cultivated mushrooms (stricter standards) and wild mushrooms (here the acceptable level for *Boletus edulis* has been further increased). Commission Regulation (EU) 2018/73 states that, due to the fact that, *inter alia*, Hg compounds are low in cultivated and wild fungi and, given the available data on their consumption in the Union, their overall contribution to dietary exposure is considered low and poses no risk to consumer health [30]. The established MRLs are considered provisional and will be reviewed in the light of information available within 10 years from the date of the Regulation’s publication. It was also noted that, in the case of Hg compounds, residues occur as a result of environmental pollution.

Considering the fact that the tested mushroom species are available for sale in large-scale chains operating in Europe supranationally (in more than one country), the range of negative effects of consuming these products is significant. The researchers addressed inquiries to representatives of the commercial networks where the mushrooms used for this study were purchased. They were asked what criteria for selecting suppliers of dried mushrooms were taken into account by a given network, whether the place of origin (type of cultivation, environment) of dried mushrooms was considered, whether the network conducted its own research on dried mushrooms offered (and if so, by what methods) and whether it verified information received from suppliers as regards the quality of dried mushrooms and their suitability for consumption. Unfortunately, none of the 5 networks provided a substantive answer to the questions (only one replied that this information was its trade secret). It seems that with such data at their disposal, networks could treat them as an asset and leverage them for commercial purposes, but there is no certainty here.

Law should minimize the risk of introducing products that may pose a threat to consumer health, especially in such a broad market as the EU. It is worth noting that before joining the European Union, Poland had stricter standards for the content of Hg, Pb and Zn in all edible mushrooms. The permissible metal content pertained to products in trade. In 2001, for unprocessed mushrooms, the standards were: Pb– 0.50 mg / kg, Cd– 0.15 mg / kg, Hg– 0.05 mg / kg, As– 0.20 mg / kg, and for dried mushrooms: Pb– 2.0 mg / kg, Cd– 1.0 mg / kg, Hg– 0.50 mg / kg, As– 0.50 mg / kg [54]. In 2003 (i.e. a year before Poland’s accession to the EU), the limits for lead in mushrooms and fresh mushroom preparations were restricted–the permissible values were 0.30 mg / kg [55].

On the basis of the above findings, we recommend that EU institutions monitor the situation related to contamination of edible mushrooms with heavy metals and consider clarifying the permissible standards of contamination in all edible mushrooms. Determining and proposing precise standards in this area requires separate and appropriately extended research.

## 4. Conclusions

The present study provides new data on the content of potentially toxic elements that, to the best of the author’s knowledge, are not referenced in the literature. The research showed that the content of all the tested toxic elements were lower in *Xerocomus badius* than in *Boletus edulis*. Estimation of health risk indexes showed that the consumption of dried mushrooms (especially *Boletus edulis*) can be associated with risk to human health resulting from intake of Hg, Cd and Pb. Finally, the results indicate that the content of toxic elements in dried wild-grown mushrooms should be monitored. The collected data justify the view that the European Union and individual Member States should clarify their regulations in order to define the maximum levels of Hg, Cd and Pb in edible mushrooms.

## Supporting information

S1 Fig Concentrations of Cd, Pb, As and Hg in dried mushrooms (μg/kg). (DOCX)

S1 Dataset (XLSX)
