# Supplementary figures and images for: Health risk assessment of exposure to toxic elements resulting from consumption of dried wild-grown mushrooms available for sale
Source: PLoS One. 2021 Jun 23;16(6):e0252834. doi: 10.1371/journal.pone.0252834 (PMC8221490; doi:10.1371/journal.pone.0252834)

| 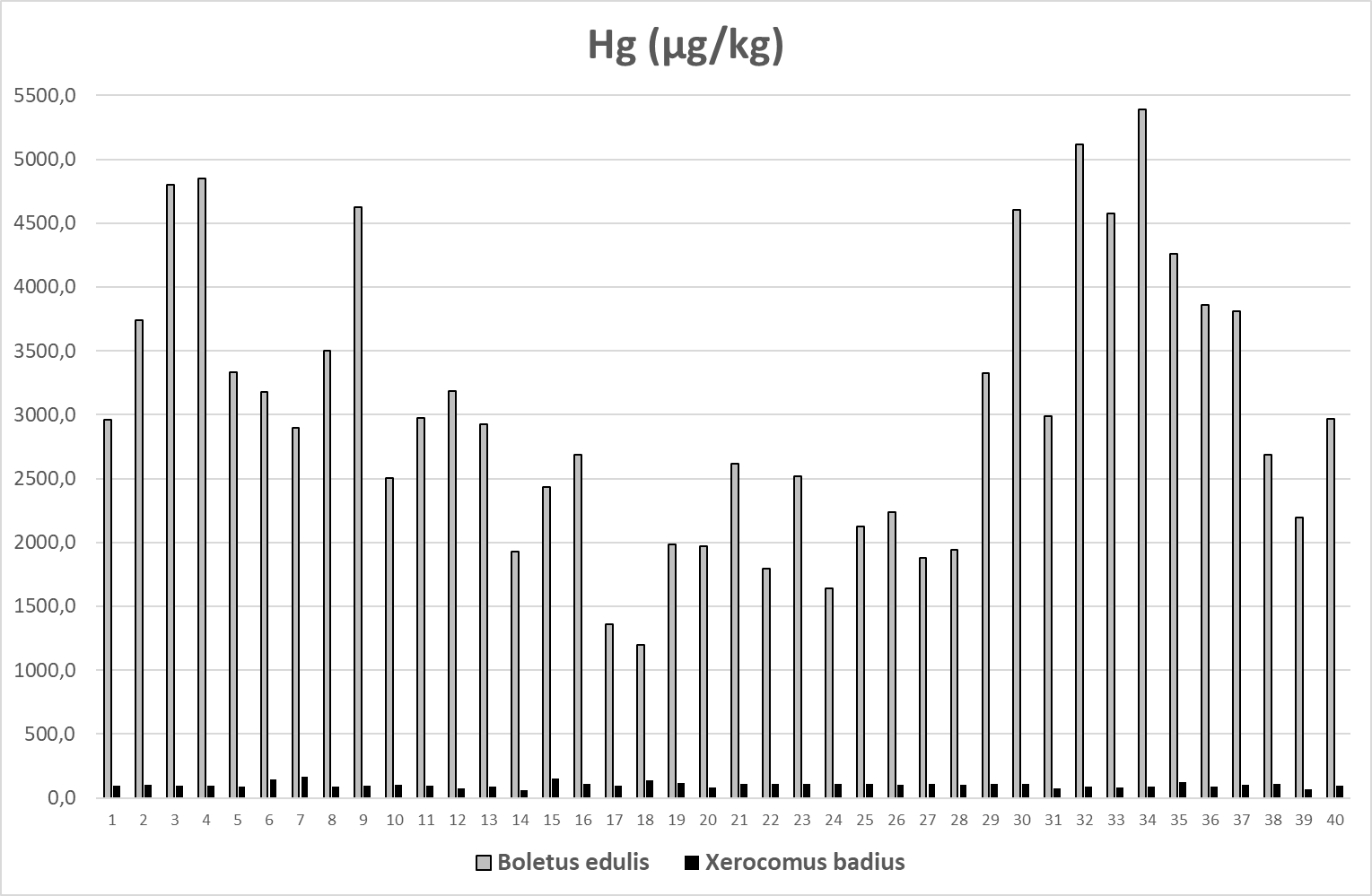 | 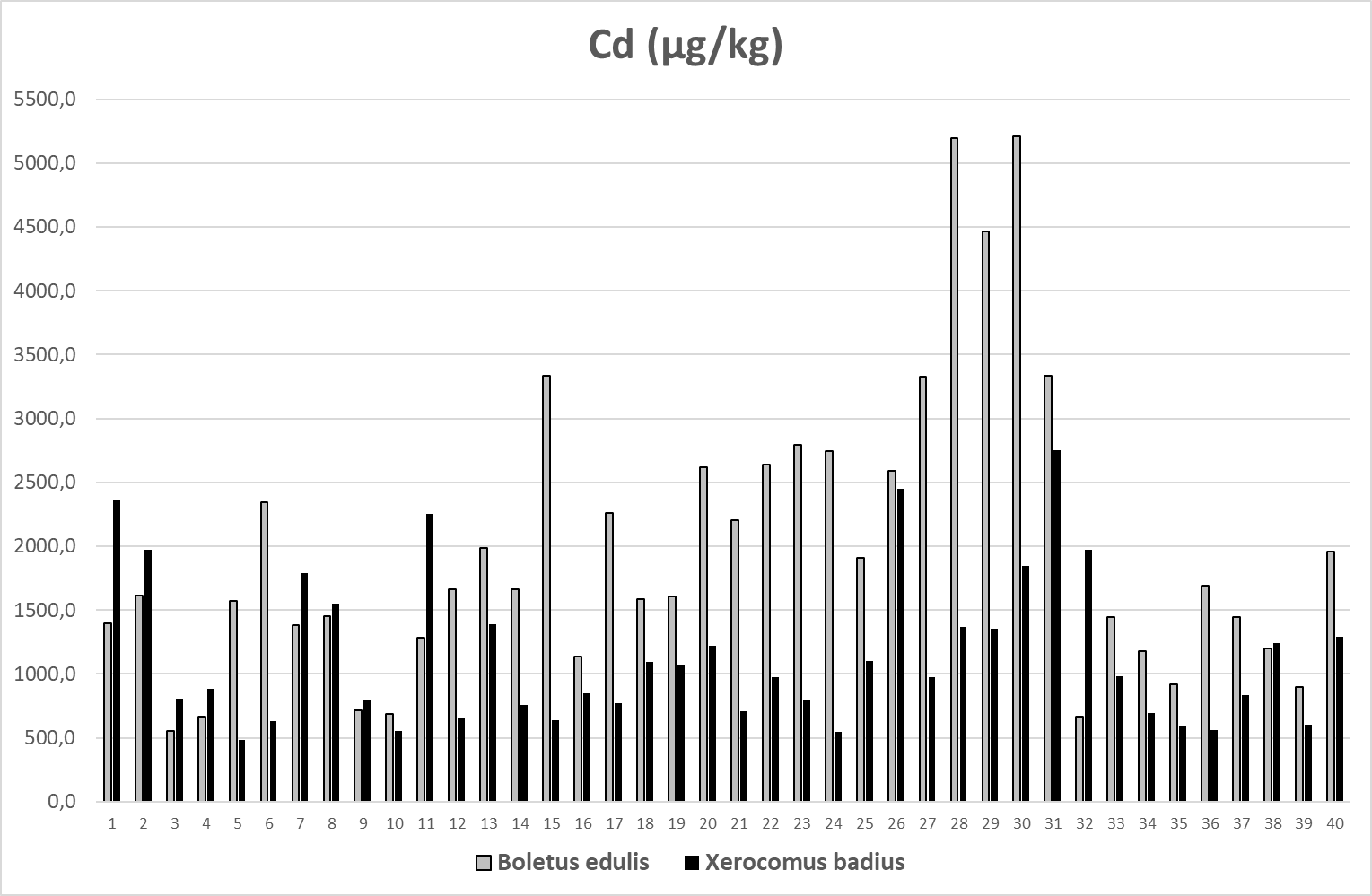 |
| --- | --- |
| 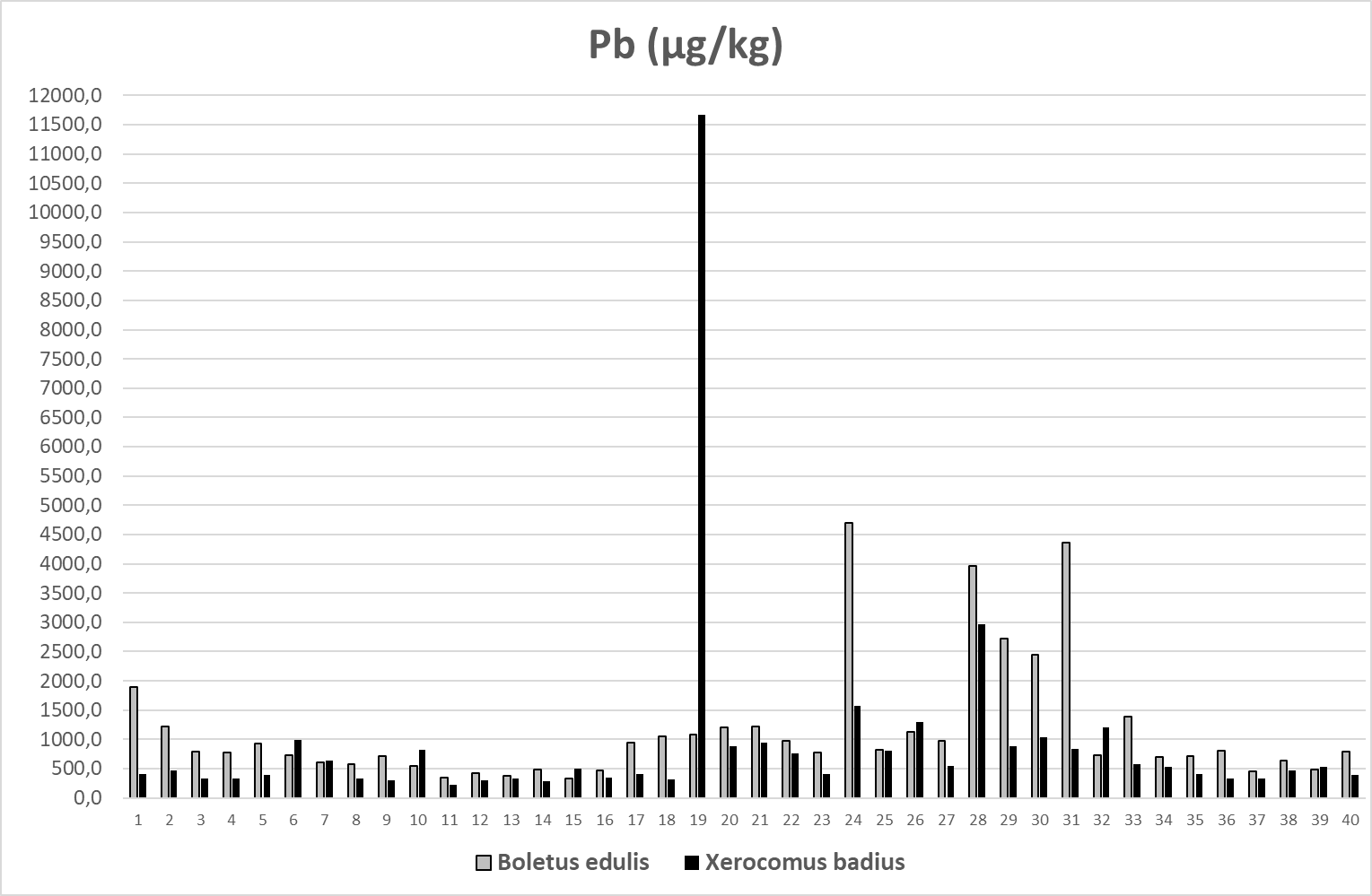 | 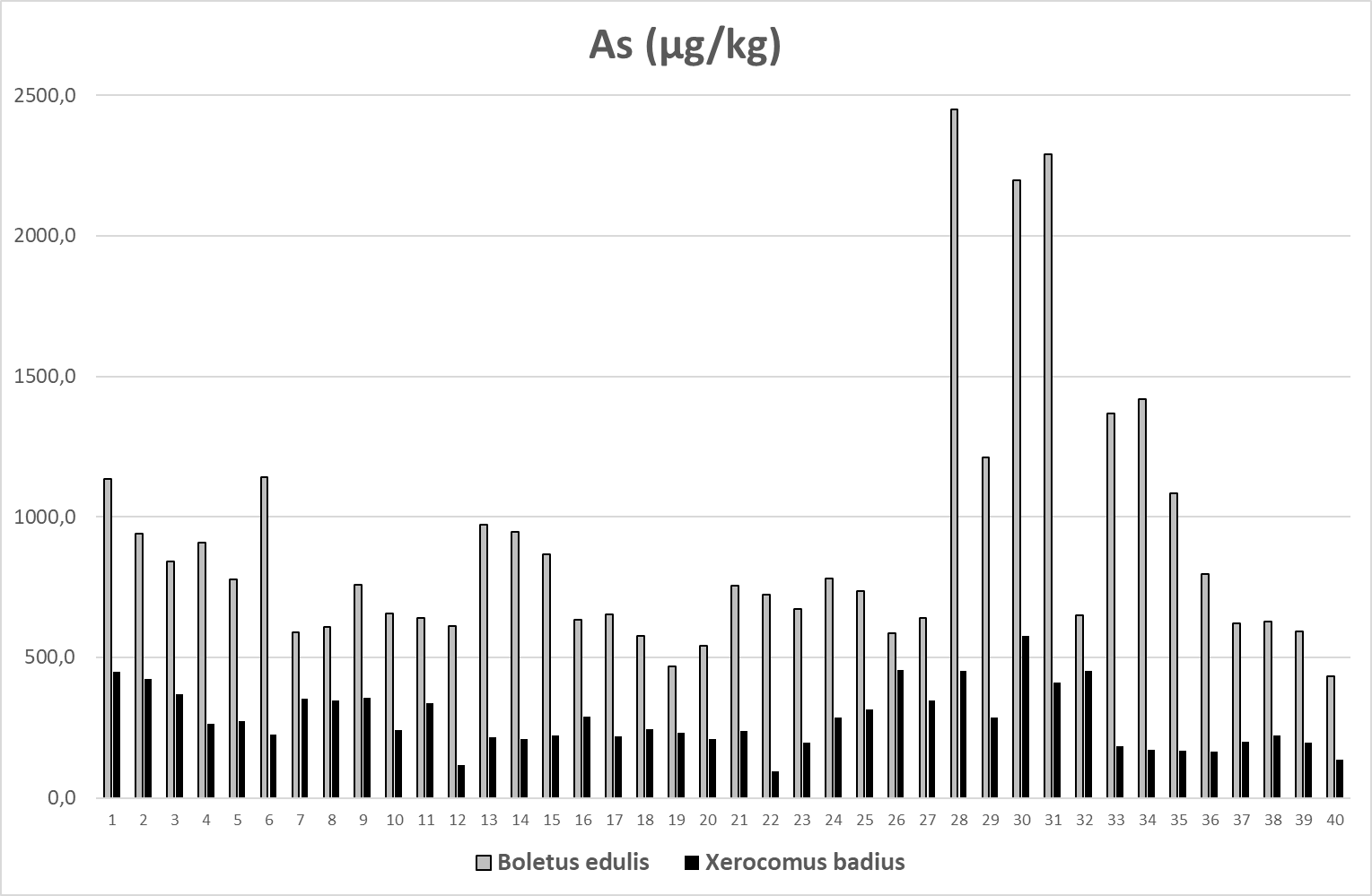 |

S1 Fig. Concentrations of Cd, Pb, As and Hg in dried mushrooms (µg/kg).

Supplement: S1 Fig — (DOCX) [file pone.0252834.s001.docx]
